# Genetic Selection for Resistance to Gastrointestinal Parasitism in Meat Goats and Hair Sheep through a Performance Test with Artificial Infection of *Haemonchus contortus*

**DOI:** 10.3390/ani11071902

**Published:** 2021-06-26

**Authors:** Yoko Tsukahara, Terry A. Gipson, Steven P. Hart, Lionel Dawson, Zaisen Wang, Ryszard Puchala, Tilahun Sahlu, Arthur L. Goetsch

**Affiliations:** 1 American Institute for Goat Research, Langston University, Langston, OK 73050, USA; terry.gipson@langston.edu (T.A.G.); steve.hart@langston.edu (S.P.H.); lionel.dawson@okstate.edu (L.D.); wzaisen@langston.edu (Z.W.); puchala@langston.edu (R.P.); tilahun.sahlu@langston.edu (T.S.); arthur.goetsch@langston.edu (A.L.G.); 2 College of Veterinary Medicine, Oklahoma State University, Stillwater, OK 74078, USA

**Keywords:** genetic selection, *Haemonchus contortus*, hair sheep, meat goats, genetic parameters

## Abstract

**Simple Summary:**

Internal parasitism has been an important constraint to small ruminant production and anthelmintic resistance has become a worldwide issue. This study evaluated a 3-year genetic selection program through activities on-farm and a centralized performance test and also provided estimates of genetic parameters of growth and response to artificial infection with *Haemonchus contortus* by goats and sheep in the southcentral USA. Considerable species as well as breed differences were found in average daily gain and response to parasite infection. Average daily gain was greater for Boer than for Kiko and Spanish goats and slightly greater for Dorper than for St. Croix sheep. Infection level (number of eggs found in feces) of Spanish and St. Croix were relatively low each year, whereas that of Kiko and Dorper was lower after selection. An indicator of anemia (packed cell volume) did not always reflect infection level, which is probably reflective of differences among animals in resilience and susceptibility to haemonchosis. Moderate to high heritabilities were found for growth performance and response to parasite infection for growing meat goat and hair sheep males under a standardized environment that suggests considerable potential for genetic improvement through selection.

**Abstract:**

Internal parasitism has been the leading cause of morbidity and mortality in small ruminants in many areas such as the southcentral USA. Among the different approaches and management practices to cope with internal parasitism, genetic selection for internal parasite resistance is recognized as one with considerable potential long-term impact. A central performance test with artificial infection of *Haemonchus contortus* for selection of growing meat goats and hair sheep for breeding to increase resistance to internal parasitism and on-farm selection of females was conducted for 3 years. The results varied considerably among breeds of goats and flocks of sheep. Spanish goats and St. Croix sheep maintained relatively low fecal egg count (FEC) each year, whereas for goats categorized as being of high resistance and Dorper sheep FEC decreased with advancing year. Packed call volume (PCV) and total serum immunoglobulin (Ig) levels were not strongly related to FEC. Genetic parameters varied between the two species, which might be related to previous selection pressure exerted for parasite resistance. Heritability of FEC was higher in goats than sheep. The genetic correlation between FEC and IgM and IgG was negative for both species, which suggests possible genetic association. Genetic and phenotypic correlations between ADG and FEC were nonsignificant for both species. In conclusion, different relationships of FEC and PCV between species require careful attention during selection and the lack of relationship between ADG and FEC suggests that selection of growing male meat goats and hair sheep for resistance to internal parasitism will not adversely affect growth performance.

## 1. Introduction

Internal parasitism has been an important constraint to small ruminant production for decades. Sheep and goats are more susceptible to some gastrointestinal parasites such as *Haemonchus contortus* than cattle. This not only limits levels and efficiencies of small ruminant production but also is sometimes prohibitive to production [1]. Especially in the southeast and southcentral USA, internal parasitism has been the leading cause of morbidity and mortality in small ruminants [2].

Many different approaches and management practices have been used to address internal parasitism, including rotational grazing, feeding nematophagus fungi to decrease pasture larvae load, treating with chemicals and other substances possessing anthelmintic properties such as condensed tannins and copper oxide wire particles, animal breeding strategies, maintaining an adequate refugia population, vaccination, etc. Treatment with commercial anthelmintic products has in the past been the primary way by which sheep and goat producers have dealt with internal parasitism. However, resistance of gastrointestinal nematodes to anthelmintics has become a very serious issue, to the extent that in many situations combinations of two or even three classes of anthelmintics given at very high dosages are ineffective [3].

Selection of small ruminants for internal parasite resistance is recognized as a potential means of coping with internal parasitism in small ruminants, with relatively more research conducted with sheep than goats [1,4]. A disciplined and stringent selection strategy is required, and potential effects on other animal conditions such as addressed with Katahdin sheep by Ngere et al. [5] and Notter et al. [6] should be evaluated.

The objectives of this study were to (1) evaluate improvement in resistance to internal parasitism that could be made through sire selection of different breeds of meat goats and hair sheep over a 3-yr period from activities on-farm and in a second generation sire performance test (on-station) in the southcentral USA and (2) estimate genetic parameters of growth and response to artificial infection with *H. contortus*.

## 2. Materials and Methods

The study occurred from 2013 to 2016 with yearly protocols approved by the Langston University Animal Care and Use Committee.

### 2.1. Second Generation Small Ruminant Central Performance Test

#### 2.1.1. Animals, Housing, and Feeding

This study involved 167 growing male goats, 210 growing male sheep, 2316 female goats, and 3442 female sheep. The animals were from six commercial farms in the southcentral USA and the American Institute for Goat Research farm at Langston University (LU), Langston, OK, USA. Collaborating farms were selected based on size or number of animals and the willingness to comply with the experimental protocol. The location, number of tested animals, year, and climate conditions of each farm are in Table 1. Farms were visited by LU personnel at 4 and 8 weeks after kidding/lambing, weaning, and 6 weeks after weaning to sample feces for determining fecal egg count (FEC) and blood for DNA profiling, determining FAMACHA© score, and obtaining kidding/lambing records. In year 1, 13 to 20 intact males (initial age: 3.8 and 5.3 mo; initial weight: 19.7 and 31.3 kg for goats and sheep, respectively) from each farm were randomly selected at weaning on the farms and transferred to a confinement facility at LU to be placed on a centralized performance test 2 weeks later. In years 2 and 3, males to be evaluated were selected from progeny of “High” and “Moderate” breeding groups described later (3.4 and 3.8 mo and 19.8 and 26.5 kg in year 2 and 4.3 and 3.9 mo and 17.8 and 28.1 kg in year 3 for goats and sheep, respectively). Upon arrival at the test site, animals walked through a foot bath with 2% chlorhexidine solution (VetOne^®^, Boise, ID, USA), were vaccinated with *Clostridiumc hauvoei*, *novyi*, *septicum*, *tetani*, *haemolyticum*, and *perfringens* types B, C, and D (Covexin 8^®^, Schering-Plough Animal Health Corp., Omaha, NE, USA), and treated for lice with pyrethroid (Cylence^®^; 1% cyfluthrin, Bayer Animal Health, Shawnee Mission, KS, USA). Animals were weighed, FAMACHA© score and body temperature were determined, blood and fecal samples were collected for initial packed cell volume (PCV) and FEC, respectively, and RFID ear tags were applied. Each herd/flock was then divided into two groups based on weight and placed in adjacent pens, with the exception of animals from Farm 6 in 2015 and 2016, which had relatively small numbers of animals.

The pens were 6.1 m × 5.6 m (i.e., 6.1 m × 1.35 m concrete with 6.1 m× 4.25 m unpaved floor areas) enclosed building. Prior to animal allocation, each pen was fully cleaned with glycolic acid (Pine-Sol^®^, The Clorox Company, Oakland, CA, USA) and disinfected with chlorhexidine 2% solution. The unpaved area was sprinkled with calcium hydroxide and covered by pine shavings for bedding. Each pen was equipped with a MK 3 FIRE automated feeding unit (Feed Intake Recording Equipment; Osborne Industries, Inc., Osborne, KS, USA). Each FIRE unit had a clear polycarbonate panel with a key hole attached to the trough that allowed only one animal to consume feed at a given time. The size of the key hole and raceway was adjusted according to animal size. Feed consumption and time of entry and exit from the feeder were recorded through use of the RFID eartags, as described by Gipson et al. [9]. There were 8 to 10 animals per pen so that daily feeder access time was not limited [10]. Pens equipped with Calan feeding gates (Calan; American Calan Inc., Northwood, NH, USA) were used for goat herds in 2013. The goats in pens with Calan gates were fitted with a collar containing an electronic key that unlocked the door of a particular gate feeder as descried by Hu et al. [11]. Each pen had an automated waterer and a trace mineralized salt block (American Stockman Big 6 Mineral Salt; 96.0–99.0% NaCl, 2400 ppm Mn, 2400 ppm Fe, 260–380 ppm Cu, 320 ppm Zn, 70 ppm I, and 40 ppm Co; Compass Minerals, Overland Park, KS, USA). The concrete area was cleaned daily and new pine shavings were periodically added.

The composition of the 50% concentrate pelletized diet, termed “Langston University Ram Test Pellet,” is shown in Table 2. Feed samples were collected every 2 weeks and analyzed for dry matter (DM), ash [12], nitrogen (Leco TruMac CN, St. Joseph, MI, USA), neutral detergent fiber with use of heat stable amylase and containing residual ash, acid detergent fiber, and acid detergent lignin (filter bag technique of ANKOM Technology Corp., Fairport, NY, USA).

#### 2.1.2. Test Procedure, Treatments, and Artificial Infection

The performance test, termed ‘Second Generation Small Ruminant Central Performance Test’ (2CPT), entailed at least a 2-wk adaptation period followed by 7 to 10 wk of data collection. The different lengths of data collection were due to the breeding schedule of each farm and prolonged deworming procedures in some instances. During adaptation, initially any animals with a beginning FEC above 0 were treated with albendazole (Valbazen^®^, Zoetis, Parsippany, NJ, USA; 20 and 10 mg/kg BW for goats and sheep, respectively) and levamisole (Prohibit^®^ Soluble Drench Powder, Agri Laboratories, Ltd., St. Joseph, MO, USA; 18 and 12 mg/kg BW for goats and sheep, respectively), given at the same time. Rumatel^®^ (19.4% morantel tartrate; Phibro Animal Health, Teaneck, NJ, USA) was also fed during the adaptation period of 2013, but was not given in subsequent years due to low palatability. There were some instances when the treatment method was not adequately effective (i.e., FEC > 600 egg per g; EPG after 8 days of the treatment) due to anthelmintic resistance noted elsewhere [3]. Therefore, on Farm 4 in 2013, 2014, and 2015 and on Farms 1 and 3 in 2014 and 2015, a more intensive treatment regime was used. This intensive regime used the same dosage as described and consisted of six Valbazen^®^ treatments 12 h apart and a Prohibit^®^ treatment at the final administration of Valbazen^®^ [13], which necessitated an extended adaptation period. After each treatment, 5 g of probiotic oral gel (Probios^®^ Bovine One Oral Gel, Menomonie, WI, USA) was given to each animal. In 2014 on Farm 5, the animals had not been placed on a pasture before the 2CPT and were assumed naïve to nematode infection. Therefore, instead of anthelmintic treatment, a dose of approximately 2000 infective L3 larvae of *H. contortus* was administered.

At 7 to 10 days after anthelmintic treatment ceased or 3 wk following the preliminary infection for animals of Farm 5 in 2014, low FEC (<600 EPG) was confirmed and animals were administered approximately 10,000 infective L3 larvae of *H. contortus* orally [14]. The larvae used were the F2 generation of the experimental susceptible pronghorn antelope strain and research farm strain that were susceptible to multiple anthelmintics and were obtained from the Veterinary Medicine and Biochemical Sciences at Texas A&M University prior to each challenge. Animals were monitored throughout the test and those with poor health conditions, such as low PCV (<20%) and feed intake, were treated and excluded from the study (Table 3). At the end of data collection, animals were treated with Valbazen and Prohibit as noted earlier and also with moxidectin (Cydectin^®^, Bayer Corporation, Pittsburgh, PA, USA; 0.5 and 0.2 mg/kg BW for goats and sheep, respectively). A FEC of 0 was confirmed 1 week later. Before being returned to the farms, animals were also subjected to a breeding soundness examination (BSE) that included determining scrotal circumference and evaluating semen motility and morphology.

### 2.2. Measures

Sample collection and measurements of PCV and FEC are described elsewhere [15]. Briefly, blood samples were collected from the jugular vein and PCV was determined at 7, 14, 21, 28, 35, 42, and 49 days after artificial infection. A blood sample at 21 days after artificial infection was also collected in a tube with ethylenediaminetetraacetic acid (BD Vacutainer^®^ EDTA tube; BD, Franklin Lakes, NJ, USA) for genomic analyses [16,17,18] and a serum collection tube without an anticoagulant (BD Vacutainer^®^ Serum tube). Serum samples were separated by centrifugation at 1500× *g* for 15 min and stored at −20 °C for immunoglobulin (Ig) analyses. Total concentrations of IgA, IgM, and IgG were determined by sandwich enzyme-linked immunosorbent assay (ELISA; Bethyl Laboratories, Inc., Montgomery, TX, USA) according to directions of the manufacturer. Whole blood was stored on Whatman^®^ FTA^®^ Mini Cards (GH Healthcare Bio-Sciences Corp., Piscataway, NJ, USA) for DNA profiling. DNA profiling (parentage and genetic marker test) was conducted at Veterinary Genetics Laboratory, School of Veterinary Medicine, University of California-Davis.

Fecal samples were collected from the rectum and FEC was determined at 21, 28, 35, 42, and 49 days after the artificial infection [14] using a modified McMaster technique [19].

Body weight was recorded in the morning every week during the test. Average daily gain (ADG) was estimated by regression. Feed intake was recorded for animals in pens with FIRE units as noted earlier but not for animals in pens with Calan gates. This was because some animals were not able to use the feeders because of their size or failing to learn how to open the feeders, in which case they were fed as a group. Residual feed intake (RFI; g/day) was calculated as described by Tsukahara et al. [20]. Briefly, RFI was determined from the difference between actual daily feed intake and expected daily feed intake estimated within herd/flock based on ADG and average metabolic body weight (BW^0.75^) during the experiment as described by Basarab et al. [21]. This calculation defines RFI variation of each animal from the group mean; therefore, the average RFI for each herd/flock was equal to zero.

### 2.3. Sire and Dam Selection and Breeding

Animals within herd/flock were categorized into three resistance groups (RG; High, Moderate, and Low) each year using the cubic clustering criterion (CCC) of SAS (SAS Institute Inc., Cary, NC, USA), which resulted in unbalanced numbers of males (Table 3). Data used were mean FEC after artificial infection, RFI (except goats in 2013), and ADG. The logarithmic transformation was used for individual mean FEC. Animals categorized in the Low-resistance group were not used for breeding, as was also the case for males that did not pass the BSE. The data and resultant RG categorization information were shared with the producers. Producers selected two to four animals in the High RG and one to three animals classed in the Moderate RG for breeding in the first and second years.

Breeding females were selected based on FAMACHA© score determined during the farm visits along with FEC at weaning. Fecal samples were collected and analyzed using the same procedure noted earlier. All females on the farms were categorized into three RG (High, Moderate, and Low) using the CCC based on the standardized (Z-score; *z = (x − µ)/σ*) FEC and FAMACHA©. The data and scores were shared with the producers. Producers selected 9 to 25 dams of the High and Moderate RG for breeding to each selected sire (i.e., High and Moderate), with avoidance of inbreeding (Table 4). Females categorized in the Low RG were not included in breeding groups.

Breeding occurred in the fall of each year. Around 2 wk after the 2CPT, assortative breeding groups were established on the farms with assistance of LU personnel. There were two to four High-resistance breeding groups and one to three Moderate-resistance breeding groups at each farm annually (Table 4). Groups were maintained in separate pastures for 6 to 8 wk on the farms. The producers provided adequate management practices including feeding, with free access to supplemental minerals and fresh clean water. In the following spring, birth date, litter size, and birth weight were recorded and identification tags were applied to each offspring. The 2CPT was repeated for three years as noted earlier.

The 2CPT conducted in this study involved different breeds of goats and sheep from various parts of the southcentral USA. In addition to the farms previously listed, there were four others (two Boer goat and two Katahdin sheep farms) that participated in 2013 and 2014 [15]; however, they were not able to continue the program in later years for various reasons and data from these farms were excluded. Therefore, Katahdin-A and Katahdin-B farms were included in the test in 2014. Consequently, the year of selection was from 2014 to 2016 for the two farms and from 2013 to 2015 for the others. There were some management practices different between 2013 and later years, such as use of Calan gates and Rumatel. However, the 2CPT was implemented in a standardized environment and the majority of procedures and on-farm activities including female selection and analysis of samples were consistent. Therefore, data presented here are in regard to relative years of 1, 2, and 3 rather than 2013, 2014, 2015, and 2016.

### 2.4. Statistical Analyses

#### 2.4.1. Breed, Year, RG

Data (i.e., ADG, RFI, and FEC for animals in the 2CPT and FAMACHA© score and FEC for females on-farm) were analyzed within herd/flock to determine RG (i.e., High, Moderate, and Low) as noted earlier and yearly effects of species, herd/flock, and RG were presented elsewhere. In this paper, the 3-yr period of the 2CPT was evaluated for breed/flock difference, RG categorization, and change in growth performance and response to artificial infection with *H. contortus* as well as genetic parameters (i.e., heritability and genetic correlation). The FEC was not normally distributed and various transformations were tested by PROC UNIVARIATE [22] for normality. A logarithmic transformation [lnFEC; ln(meanFEC + 100)] was most appropriate and selected for the analyses, and results are shown in the original scale. Performance data of goat sire candidates that had completed the 2CPT (n = 144) were analyzed by PROC MIXED of SAS [23] with fixed effects of breed, year, RG, and their interactions, and the random effect was animal within breed × RG. Initial values of FEC and PCV at the time of artificial infection were used as covariates. Instead of breed, flock was used for data of growing sheep males (n = 190) because of the two farms of the Katahdin breed. Initially, effects of breeding groups were anticipated, but correlations between sires and progeny in mean FEC were not significant for any of the breeds and flocks and, thus, were not considered in the statistical analysis.

#### 2.4.2. Genetic Parameter Estimates

Genetic parameters for ADG, mean lnFEC, mean PCV, IgA, IgM, and IgG levels were estimated by the average information restricted maximum likelihood (AIREML) using WOMBAT [24] with a multivariate animal model for each species. The additive genetic and residual effects were assumed to be normally distributed with means of 0. The multivariate linear mixed model for the six traits was

*y* = *Xβ* + *Zu* + *e*,

where *y* is the vector of observations, *X* is the incidence matrix, *β* is the fixed effects (i.e., breed and year), *Z* is the incidence matrix, *u* is the random effect (i.e., animal), and *e* is the residual error. The vector *u* represents the additive genetic effect of $A\sigma_{a}^{2}$, where *A* is the additive relationship matrix and $\sigma_{a}^{2}$ is the direct additive variance component. Vector *e* represents the residual effects $I\sigma_{e}^{2}$, where *I* is an identity matrix with the dimension of the number of animals and observations and $\sigma_{e}^{2}$ is the residual variance component. Phenotypic variance ($\sigma_{p}^{2}$) was the sum of the direct additive and residual variance. Heritability (*h^2^*) of each trait was the ratio of additive genetic portion to phenotypic variance ($\sigma_{a}^{2}/\sigma_{p}^{2}$). Genetic and phenotypic correlations were the ratio of a covariance component of the pairwise bivariate (i.e., the two traits for the same animals) to the square root of their variances also calculated by WOMBAT [24]. The two-tailed Student t-distribution was used for hypothesis testing and calculation of *P* values. There were 147 observations for growing male goats and 200 for sheep males. The pedigree record consisted of 147 and 200 animals, 32 and 57 known sires, 95 and 152 known dams with 4 and 10 full-sibs, and 97 and 149 half-sibs for goats and sheep, respectively. The number of observations was greater than the analysis using the MIXED model because males that were not selected for breeding due to BSE failure were included in this analysis.

## 3. Results

### 3.1. Goats

There were many significant two-way interactions (Table 5 and Table 6), which will be addressed. However, because main effects also are of interest, main effect means are presented for each variable. For ADG, there were breed × year and breed × RG interactions (*p* < 0.05). Overall, ADG ranked (*p* < 0.05) Boer > Kiko > Spanish, although the lower value for Spanish than for Kiko was because of no increase in BW in year 1, with values in years 2 and 3 not different from those of Kiko (*p* > 0.05). The main effect of RG on ADG was lowest for Low (*p* < 0.05). However, there were some RG differences that varied among the breeds.

There were breed × year, breed × RG, and year × RG interactions in mean FEC (*p* < 0.05). Spanish goats had low and similar FEC each year, whereas for Kiko FEC was lower in year 2 and 3 than in year 1 (*p* < 0.05). The FEC for the High RG was lowest among breeds for Spanish (*p* < 0.05), but values were similar among breeds for Moderate and Low RG. Moreover, FEC for the High RG was much lower in year 2 and 3 than in year 1 (*p* < 0.05), although values were similar among years for Moderate and Low RG. Mean PCV was influenced by an interaction (*p* < 0.05) between breed and year. Overall, PCV ranked year 1 < 3 < 2 and was greatest among RG for High (*p* < 0.05). The primary factor responsible for the breed × year interaction was a relatively high value for Kiko in year 2 (*p* < 0.05).

There were breed × year interactions (*p* < 0.05) in concentrations of IgA, IgM, and IgG (Table 5 and Table 6), with significant differences among breed main effects for IgM and IgG (*p* < 0.05) and among years for each of the Ig (*p* < 0.05). Levels of each Ig were considerably greater in year 3 than in earlier years (*p* < 0.05). The difference between the IgA concentration in year 3 vs. years 1 and 2 was greater for Kiko than for Boer and Spanish (*p* < 0.05). Main effect means for IgM and IgG were lower for Kiko than for Boer and Spanish (*p* < 0.05). The factor that appeared most responsible for the interactions in IgM and IgG levels increased from year 1 to 2 and from year 2 to 3 for Kiko in contrast to the fairly similar values for Boer and Spanish in years 1 and 2 with major change only from year 2 to 3.

### 3.2. Sheep

As for goats, there were numerous significant interactions as well as main effects for sheep (Table 7 and Table 8). The ADG was greater for Dorper and Katahdin-A than for Katahdin-B and St. Croix (*p* < 0.05) and in year 1 vs. year 2 and 3 (*p* < 0.05). The primary factor responsible for the flock × year interaction (*p* < 0.05) was higher values in year 1 for Katahdin-B and St. Croix than in year 2 and 3 in contrast to more similar values among years for Dorper and Katahdin-A. Mean FEC ranked (*p* < 0.05) St. Croix < Katahdin-A < Dorper and Katahdin-B and was lowest among years in year 3 (*p* < 0.05). There was a significant three-way interaction in FEC (*p* = 0.047), with two-way interactions presented in Table 8. The FEC for Dorper decreased each year in contrast to more similar values among years for Katahdin-A and St. Croix, with a value in year 2 for Katahdin-B much greater than in year 1 and 3. The FEC for High RG was much less for Katahdin-A and St. Croix than for Dorper and Katahdin-B (*p* < 0.05), and the level for the Low RG of St. Croix was less than for Dorper, Katahdin-A, and Katahdin-B (*p* < 0.05). The PCV ranked (*p* < 0.05) St. Croix > Dorper > Katahdin-A and Katahdin-B and RG High > Moderate > Low and was greatest among years for year 1 (*p* < 0.05). One factor contributing to the flock × year interaction showed greater differences among years for Katahdin-A compared with other flocks. Moreover, for the year × RG interaction, differences among years were less for the High RG than for Moderate and Low.

The IgA concentration ranked (*p* < 0.05) St. Croix > Katahdin-A > Dorper and Katahdin-B, was greatest among years in year 1 (*p* < 0.05), and was greater for the High than Moderate RG (*p* < 0.05; Table 7 and Table 8). The high value for St. Croix is attributable to year 1 and 2 concentrations for Katahdin-A and Katahdin-B that were similar among years (*p* > 0.05) in contrast to Dorper and St. Croix. The IgM concentration was greater for Katahdin-B and St. Croix than for Dorper and Katahdin-A (*p* < 0.05), and differences among years varied considerably among flocks. The IgG concentration was lower in year 1 vs. year 2 and 3 (*p* < 0.05), with relatively smaller differences among years for Katahdin-A compared with other flocks.

### 3.3. Genetic Parameters

Summarized statistics for ADG, FEC, PCV, and Ig concentrations used to estimate genetic variance components are presented in Table 9. Average daily gain was greater for sheep than for goats, although variability was greater for goats as assessed by the coefficient of variation (CV; ratio of SD to the mean). The PCV was numerically lower for goats than for sheep. The CV of IgA concentration for sheep was very high but that of other traits were similar between goats and sheep.

The multivariate analysis for goat and sheep datasets converged based on three criteria (i.e., the increase in log likelihood values, change in the vector of parameter estimates, and the norm of the gradient vector) [24]. Estimates of additive genetic, residual, and phenotypic variance and heritability for each trait are shown in Table 10. Heritability for ADG was moderate for goats and very high for sheep that had much lower residual variance. Heritabilities of FEC and PCV were greater for goats but those of IgA, IgM, and IgG were greater for sheep than for goats.

Genetic and phenotypic correlations between traits for growing male goats and sheep are presented in Table 11. There were many significant genetic correlations for both goats and sheep. The ADG was positively correlated (*p* < 0.01) with PCV and negatively correlated (*p* ≤ 0.01) with IgM and IgG for goats and sheep. The correlation between ADG and IgA was positive (*p* < 0.01) for goats but was negative (*p* < 0.01) for sheep. The FEC was negatively correlated (*p* < 0.01) with PCV for goats but the correlation was positive (*p* < 0.01) for sheep. The FEC was positively correlated (*p* < 0.01) with IgA for goats but not for sheep. Negative correlations (*p* < 0.01) between FEC and serum IgM and IgG were found for both goats and sheep. Both species had positive correlations (*p* < 0.01) between PCV and IgA and negative correlations (*p* ≤ 0.03) between PCV and IgG. The IgA was negatively correlated (*p* < 0.01) with IgM and IgG for goats but those relationships were positive (*p* < 0.01) for sheep. High and positive correlation was found between IgM and IgG for both species.

Phenotypic correlations were much less significant than genetic correlations. The ADG was negatively correlated (*p* < 0.01) with IgM and IgG for goats but it was not correlated (*p* = 0.13) with IgM for sheep. The correlation between FEC and PCV was not significant (*p* = 0.06) for goats but was negative (*p* < 0.01) for sheep. The FEC was negatively correlated (*p* = 0.01) with IgG for goats but the correlation for sheep was not significant (*p* > 0.06). Serum IgA, IgM, and IgG concentrations were positively correlated (*p* ≤ 0.02) for goats, but for sheep the only positive correlation was between IgM and IgG (*p* < 0.01).

## 4. Discussion

### 4.1. Second Generation Small Ruminant Central Performance Test (2CPT)

Genetic selection for resistance to internal parasitism, such as infection with *H. contortus*, is one of the practical and ecologically friendly strategies to address the issue. Some other strategies such as maintenance of a high nutritional plane, feeding condensed tannin-rich legumes, and rotational grazing would not always be economically feasible or practical. Culling susceptible animals would minimize or prevent pasture contamination, lessen the prevalence of genes in the population contributing to susceptibility, and avert overuse of available anthelmintics to slow the increase in internal parasite resistance until better targeted strategies such as potential ones of genome editing and use of the microbiome become available.

There is no direct measurement of small ruminant resistance to infection with *H. contortus*, but the most common method of FEC was assessed in this study. Packed cell volume and FAMACHA© score are also commonly used as indicators of haemonchosis, which causes anemia in small ruminants. Change in blood immunoglobulin (i.e., IgA, IgM, and IgG) concentrations in small ruminants in response to internal parasite infection seems promising for use in selection to increase resistance to helminths [1], although the levels of each Ig can vary with species, breed, age, measurement method, etc., [25].

### 4.2. On-Farm Activities and Conditions

On-farm data collection was conducted by LU personnel, with the same individuals responsible for specific activities such as determining the FAMACHA© score, data recording, blood and fecal sampling, and FEC analysis to avoid personnel variance. Establishing breeding groups on the larger farms in the study was relatively easy, and based on activities in year 1 more females were included in the second year of breeding. However, the relatively small Katahdin-B farm experienced wet conditions in year 2 (2015) and lost some females; hence, many females in the breeding groups were replaced.

### 4.3. Growth Performance

As expected, ADG varied among breeds, especially for goats, because of different characteristics of the breeds. Although the three breeds of goats are categorized as “meat,” Boer goats have been selected for high growth rate [26], and Kiko goats have been selected for survivability and growth rate in harsh environments [27]. Spanish goats are also known for their tolerance to harsh conditions, as Blackburn [28] indicated that growth rate and fecundity may be greater than for Boer with low-quality forage consumption. Slightly lower ADG for St. Croix than for Dorper is in accordance with differences in mature size and what the breeds have been selected for [29,30]. Lower overall ADG for Katahdin-B vs. Katahdin-A, with similar values for Dorper and Katahdin-A, was because of low ADG of Katahdin-B in year 2, again relating to the poor initial condition of the males due to wet conditions in 2015 at that farm.

The difference in overall ADG among RG of goats in contrast to similar values for sheep may reflect relatively greater impact of internal parasitism on performance of goats than sheep [31,32]. Although, the interaction between goat breed and RG suggests that this could be a more important factor for Boer and perhaps Kiko than for Spanish goats. The results likewise support a conclusion that selecting for resistance to internal parasitism, which conceivably could increase nutrient and energy use for associated immune responses to infection, did not adversely affect the growth performance of either goats or sheep.

Heritability of ADG of goats of 0.484 was greater than 0.18–0.26 estimated by Schoeman et al. [33] for Boer goats preweaning and 0.23–0.35 by Dige et al. [34] for Jamunapari goats. However, differences in ADG heritability estimated in this study and previous reports were much greater in sheep. The heritability of 0.847 is in contrast to 0.14–0.21 for a mutton breed of India [35] and 0.12–0.30 for Dorper sheep in Kenya [36]. Reasons for the relatively high estimates in the current study include the low residual variance primarily due to a standardized environment (i.e., same feed and feeding regime), especially compared with the 30 years of observation data in India [35] and data of grazing animals in two pastures in Kenya [36].

### 4.4. FEC and PCV

Fecal egg count and PCV are common indicators of *H. contortus* infection, and both varied among species, breed, flock, and year as well as RG. The two species had similar mean FEC (1669 and 1639 EPG for goats and sheep, respectively; *p* = 0.850), but PCV was lower in goats (26.0 vs. 29.0%; *p* < 0.001). Lower PCV for goats can be partially explained by the smaller erythrocytes of goats than sheep, which results in tighter packing upon centrifugation [37].

The significant interaction in FEC between RG and year for goats but not sheep may reflect greater susceptibility to internal parasitism of goats as noted earlier and, thus, greater potential for increased resistance with selection. It is notable that for goats there was an appreciable decrease in FEC of High RG animals from year 1 to year 2 and 3 converse to no differences among years for the Moderate RG and also for the Low RG. Although the three-way interaction in FEC involving breed, year, and RG was not significant, the lower breed × RG mean of the High RG for Spanish vs. Boer and Kiko suggests greater potential for improved resistance of these two breeds. Applying a similar analogy to sheep would imply greater potential for improved resistance of Dorper vs. St. Croix and a considerable difference between the two Katahdin farms. In support, St. Croix is known as a breed with relatively high resistance to infection with *H. contortus* [30]. The difference in FEC between Katahdin-A and Katahdin-B brings out an obvious and important point, which is that other than for the Katahdin sheep breed, there was one farm per breed. That is, farm is confounded with breed. Certainly there would be interest in comparing the different species and breeds under similar conditions. However, to do so conceivably would disadvantage some groups perhaps not as well adapted to or suited for these common conditions. Having more than one farm per breed would be desirable, but from a practical and realistic standpoint this is challenging when working with individual farms and real-world conditions with small ruminants raised for economic returns. Although, in some situations with very low numbers of animals per farm, such as addressed by Goetsch [38] regarding on-farm research in developing countries, this would be a more feasible consideration.

Heritabilities of mean FEC and mean PCV estimated in this study were higher than 0.07 and 0.22 for dairy goats [39], a closed line of Kiko × Boer goats selected for internal parasite resistance (0.13 and 0.06) [40], and Creole goats (0.14–0.37 and 0.10–0.33 for FEC and PCV, respectively) [41]. The FEC heritability of sheep in this study was within the range of reported values, 0.18–0.46 for Katahdin lambs with natural infection [5], 0.19–0.60 for Merino sheep with natural infection [42], and 0.10–0.19 for lambs and 0.31 for ewes of a composite breed with artificial infection [4]. The heritability of PCV in this study was between 0.15 for ewes and 0.39 for lambs estimated by Vanimisetti et al. [4].

Reports of genetic correlations for goats between FEC and PCV of −0.41 [39], −0.06 to −0.67 [41], and −0.53 [43] are all similar to the estimate in this study. This could reflect comparable effects of genetic selection on these two indicators in goats. Conversely, the genetic correlation between PCV and FEC was positive for sheep, whereas the phenotypic correlation was negative. Álvarez et al. [44] reported a nonsignificant genetic correlation between FEC and PCV of sheep with a bivariate model (−0.038–0.181) but a significant and positive correlation with a trivariate model (0.432–0.695).

Phenotypic correlations between FEC and PCV in this study were not significant for goats but were negative for sheep. Tsukahara at al. [15] reported that the phenotypic relationship of the two indicators varied also among breeds of goats and sheep. These findings suggest that PCV may not always reflect FEC, which may relate to variation in tolerance or resilience to *H. contortus*.

### 4.5. Immune Response

The lack of significant effect of RG on Ig concentrations in goats and nonsignificant interactions involving RG are not supportive of strong relationships to changes in resistance to internal parasitism during the study period. Findings for sheep were fairly similar, as might be expected given the lack of differences in FEC among RG. Although helminth-specific Ig were not measured, there was a difference among RG in IgA, with a lower value for Moderate than for Low.

In accordance with the general lack of effect of RG on Ig, phenotypic correlations between mean FEC and serum IgA, IgM, and IgG levels were not significant or strong for goats or sheep except for the correlation between FEC and IgG for goats. There were again breed differences. Conversely, genetic correlations between FEC and Ig were significant for goats and sheep except for the correlation between FEC and IgA for sheep. Estrada-Reyes et al. [17] reported SNP associated with FEC, ADG, and IgM in goats, which supports possible genetic selection for both growth and resistance to haemonchosis. Further research is required to characterize relationships between haemonchosis indicators (i.e., FEC and PCV) and serum Ig concentrations.

### 4.6. Discussion Summary

A central performance test with artificial infection of *H. contortus* for selection of growing meat goats and sheep for breeding to increase resistance to internal parasitism and on-farm selection of females was conducted for 3 years. The results varied considerably among breeds of goats and flocks of sheep. Spanish goats and St. Croix sheep maintained relatively low FEC each year, whereas FEC for Kiko and Dorper was lower in year 2 and(or) 3 than in year 1, suggesting increased resistance to internal parasitism due to selection. Moreover, for goats FEC was lower in year 2 and 3 than in year 1 for animals categorized as being of high resistance, with those tested in year 2 and 3 being progeny of sires classed similarly. Conversely, there were no differences in FEC among years for animals classed as being of moderate or low resistance. In contrast, and perhaps in agreement with sheep being more resistant than goats and only a decrease in FEC for Dorper with advancing year, there were no effects of year or year × resistance group interactions with the four hair sheep flocks evaluated. The PCV and serum immunoglobulin levels were not always strongly related to FEC.

## 5. Conclusions

Genetic improvement from selecting goats and sheep in the resistance to internal parasitism and growth performance was evaluated under a standardized environment. Resistance and response to selection varied considerably among species, breeds, and farms with the same breed. Genetic parameters also varied between the two species, which might be related to previous selection pressure exerted for parasite resistance. Nonetheless, moderate to high heritabilities were found for growth performance and response to artificial infection with *H. contortus*, which suggests considerable potential for genetic improvement through selection. Selection of growing male meat goats and hair sheep for resistance to internal parasite infection did not adversely affect the growth performance.

## Figures and Tables

**Table 1:** Species, breed, location, and production type of farms and number of animals tested and climate by year.

								Climate				
						Tested Animals ^1^		Average Temperature ^2^, °C				
Farm	Species	Breed	State	Production Type	Year Tested	Male (in 2CPT)	Female (Kid/Lamb)	Mean	Maximum	Minimum	Average Humidity ^2^, %	Total Precipitation ^3^, cm
1	Goat	Boer	Oklahoma	Research	2013	103 (20)	265 (61)	15.5	21.6	9.8	64.6	103.1
					2014	70 (18)	200 (58)	15.5	21.8	9.2	59.9	44.9
					2015	26 (16)	82 (29)	16.3	22.5	10.8	66.6	94.7
2	Goat	Kiko	Kansas	Commercial	2013	149 (18)	448 (169)	13.0	18.8	7.4	71.7	151.7
					2014	140 (17)	308 (119)	11.7	18.4	5.0	66.3	105.2
					2015	211 (21)	473 (203)	14.3	20.2	8.6	70.2	76.8
3	Goat	Spanish	Oklahoma	Research	2013	119 (19)	241 (109)	15.5	21.6	9.8	64.6	103.1
					2014	56 (18)	171 (50)	15.5	21.8	9.2	59.9	44.9
					2015	42 (20)	128 (44)	16.3	22.5	10.8	66.6	94.7
4	Sheep	Dorper	Missouri	Commercial	2013	69 (20)	241 (63)	12.8	18.4	7.7	71.3	120.4
					2014	101 (20)	312 (133)	12.8	18.6	7.3	68.0	105.0
					2015	92 (20)	346 (138)	14.1	19.7	9.1	70.1	169.8
5	Sheep	Katahdin	Missouri	Commercial	2014	213 (20)	606 (183)	13.4	19.0	7.7	68.0	81.0
					2015	279 (20)	628 (244)	14.7	20.3	9.5	71.0	148.4
					2016	252 (21)	595 (234)	15.6	21.3	10.3	70.2	100.3
6	Sheep	Katahdin	Arkansas	Commercial	2014	41 (18)	131 (33)	13.9	20.1	8.5	75.3	120.7
					2015	14 (11)	76 (27)	15.3	21.4	10.0	74.0	175.6
					2016	13 (8)	112 (67)	16.8	23.2	11.1	76.8	127.9
7	Sheep	St. Croix	Missouri	Commercial	2013	19 (13)	124 (24)	12.4	18.1	7.1	71.2	133.8
					2014	34 (19)	160 (66)	12.4	18.1	6.9	68.6	84.6
					2015	33 (20)	111 (34)	13.9	19.5	8.7	70.3	146.7

^1^ 2CPT = second generation central performance test. ^2^ Weather underground [7] at stations near farms. ^3^ US climate data [8] at stations near the farms.

**Table 2:** Ingredients and chemical composition of the pelletized diet fed to growing male goats and sheep.

Item	Concentration	
Ingredient, % (as fed basis)		
Dehydrated alfalfa	19.98	
Cottonseed hulls	29.07	
Cottonseed meal	8.99	
Ground corn	19.98	
Wheat middlings	12.99	
Pelletizing agent	4.99	
Salt	1.00	
Calcium carbonate	0.95	
Ammonium chloride	1.00	
Yeast	1.00	
Vitamin-mineral mixture ^1^	0.05	
Rumensin^®^ 90 premix ^2^	0.01	
Chemical composition ^3^	Mean	SEM
Ash, % of DM	7.8	0.12
Crude protein, % of DM	17.2	0.17
Ether extract, % of DM	2.0	0.04
Neutral detergent fiber, % of DM	42.3	0.51
Acid detergent fiber, % of DM	38.4	2.00
Acid detergent lignin, % of DM	9.1	0.16

^1^ Contained 1.28% Zn, 0.96% Fe, 0.704% Mn, 0.16% Cu, 0.048% I, 0.032% Co, 26,460,000 IU/kg vitamin A, 6,615,000 IU/kg vitamin D_3_, and 11,025 IU/kg vitamin E as fed basis. ^2^ 20% monensin for coccidian control. ^3^ 74 samples, collected every other week during tests from 2013 to 2016.

**Table 3:** Number of growing male goats and sheep in resistance groups classified by the cubic clustering criterion by year.

			Resistance Group			
Farm	Breed/Flock	Year	High	Moderate	Low	Excluded ^1^
1	Boer	1	6	5	5	4
		2	3	6	3	6
		3	4	5	7	0
2	Kiko	1	8	6	2	2
		2	7	4	3	3
		3	6	11	3	0
3	Spanish	1	5	5	4	5
		2	7	7	3	1
		3	4	6	9	1
4	Dorper	1	8	9	3	0
		2	8	5	2	5
		3	11	4	4	1
5	Katahdin-A	1	4	7	6	3
		2	9	7	1	3
		3	7	11	3	0
6	Katahdin-B	1	9	5	4	0
		2	3	3	2	3
		3	2	2	4	0
7	St. Criox	1	8	2	3	0
		2	5	5	4	5
		3	7	9	4	0

^1^ Animals excluded from sire selection due to health issues or not passing the breeding soundness evaluation.

**Table 4:** Number of female goats and sheep in breeding groups ^1^ for each farm in the first and second years.

			High Resistance Group				Moderate Resistance Group		
Farm	Breed/Flock	Year	1	2	3	4	1	2	3
1	Boer	1	17	16	16		14	14	15
		2	13	12	13		13	12	12
2	Kiko	1	16	15	14		15	15	15
		2	15	15	15	15	13	13	
3	Spanish	1	14	14	14		15	14	14
		2	13	14	15		15	15	15
4	Dorper	1	11	11	12		12	12	11
		2	14	15	17		15	15	15
5	Katahdin-A	1	15	15	15		15	15	15
		2	16	16	17	16	15	16	17
6	Katahdin-B	1	14	13	9		13	13	
		2	19	17			13		
7	St. Croix	1	10	13			14	11	
		2	25	22			13	11	

^1^ Females were selected based on on-farm data and groups were determined by the producers.

**Table 5:** *p* values for effects of breed, year, resistance group, and their interactions on growth and response to artificial infection with *Haemonchus contortus* by growing male goats.

	Source of Variation ^1^						
Item ^2^	Breed	Year	Breed × Year	RG	Breed × RG	Year × RG	Breed × Year × RG
ADG, g/day	<0.001	<0.001	<0.001	0.017	0.039	0.341	0.771
Mean lnFEC	<0.001	0.018	<0.001	<0.001	<0.001	0.005	0.497
Mean PCV, %	0.133	<0.001	0.001	<0.001	0.104	0.197	0.511
IgA, mg/L	0.061	<0.001	0.041	0.812	0.443	0.075	0.690
IgM, mg/L	<0.001	<0.001	<0.001	0.764	0.082	0.738	0.704
IgG, g/L	<0.001	<0.001	<0.001	0.773	0.234	0.597	0.933

^1^ Breed: Boer, Kiko, and Spanish; Year: 1, 2, and 3; RG (Resistance group): High, Moderate, and Low. ^2^ ADG = average daily gain; lnFEC = logarithmic transformed fecal egg count [log(meanFEC + 100)]; PCV = packed cell volume.

**Table 6:** Effects of breed, year, and resistance group on growth and response to artificial infection with *Haemonchus contortus* by growing male goats.

		Breed				Year				Resistance Group			
Item ^1^	Year, RG ^2^	Boer	Kiko	Spanish	SEM	1	2	3	SEM	High	Moderate	Low	SEM
ADG, g		242 ^a^	150 ^b^	90 ^c^	7.7	130 ^b^	181 ^a^	172 ^a^	7.7	166 ^a^	174 ^a^	142 ^b^	7.65
	1	235 ^a^	156 ^b^	−1 ^c^	13.3								
	2	254 ^a^	158 ^b^	130 ^b^									
	3	238 ^a^	138 ^b^	141 ^b^									
	High	247 ^ab^	141 ^d^	110 ^ef^	13.2								
	Moderate	267 ^a^	177 ^c^	79 ^f^									
	Low	213 ^b^	134 ^de^	81 ^f^									
Mean FEC, eggs/g		1603 ^b^	2078 ^b^	1375 ^a^	133.8	2043 ^b^	1243 ^a^	1770 ^ab^	132.9	840 ^a^	1553 ^b^	2663 ^c^	133.0
	1	1046 ^a^	3781 ^c^	1303 ^a^	230.8								
	2	1134 ^a^	1239 ^a^	1357 ^a^									
	3	2630 ^b^	1216 ^a^	1464 ^a^									
	High	822 ^b^	1381 ^b^	318 ^a^	229.1	1359 ^b^	610 ^a^	552 ^a^	229.2				
	Moderate	1377 ^c^	1962 ^c^	1320 ^c^		1951 ^cd^	1268 ^c^	1440 ^cd^					
	Low	2611 ^d^	2892 ^d^	2487 ^d^		2821 ^e^	1852 ^de^	3318 ^e^					
Mean PCV, %		25.9	26.6	25.7	0.31	24.9 ^c^	27.2 ^a^	26.0 ^b^	0.11	27.1 ^a^	25.6 ^b^	25.5 ^b^	0.31
	1	24.7 ^c^	25.0 ^c^	25.0 ^c^	0.56								
	2	26.7 ^b^	29.3 ^a^	25.7 ^bc^									
	3	26.2 ^b^	25.5 ^bc^	26.4 ^b^									
IgA, µg/mL		39.1	33.2	40.5	2.25	31.7 ^b^	32.7 ^b^	48.3 ^a^	2.25	37.2	38.7	36.9	2.24
	1	36.0 ^c^	24.5 ^d^	34.7 ^cd^	3.89								
	2	34.8 ^cd^	23.5 ^d^	39.9 ^bc^									
	3	46.5 ^ab^	51.7 ^a^	46.8 ^ab^									
IgM, µg/mL		1060 ^a^	817 ^b^	1181 ^a^	44.2	766 ^b^	890 ^b^	1401 ^a^	44.2	1045	1006	1007	44.0
	1	860 ^cd^	409 ^e^	1030 ^bc^	76.4								
	2	821 ^cd^	685 ^d^	1164 ^b^									
	3	1499 ^a^	1358 ^ab^	1347 ^ab^									
IgG, mg/mL		9.6 ^a^	7.8 ^b^	9.5 ^a^	0.35	7.1 ^b^	7.7 ^b^	12.2 ^a^	0.35	9.2	8.9	8.9	0.35
	1	8.7 ^b^	3.9 ^d^	8.6 ^bc^	0.61								
	2	7.5 ^b^	7.0 ^c^	8.5 ^bc^									
	3	12.7 ^a^	12.4 ^a^	11.4 ^a^									

^1^ ADG = average daily gain, FEC = logarithmic transformed fecal egg count [log(meanFEC + 100)] was used for analysis and results were presented in the original scale, PCV = packed cell volume; IgA, IgM, and IgG = immunoglobulin A, M, and G, respectively; ^2^ RG = resistance group; ^a–f^ Means within a grouping (i.e., breed, year, breed × year, resistance group, breed × resistance group, year × resistance group) without a common superscript letter differ (*p* < 0.05).

**Table 7:** *p* values for effects of flock, year, resistance group, and their interactions on growth and response to artificial infection with *Haemonchus contortus* by growing male sheep.

	Source of Variation ^1^						
Item ^2^	Flock	Year	Flock × Year	RG	Flock × RG	Year × RG	Flock × Year × RG
ADG, g	<0.001	<0.001	0.012	0.354	0.992	0.155	0.205
Mean lnFEC	<0.001	0.060	<0.001	<0.001	<0.001	0.639	0.047
Mean PCV, %	<0.001	<0.001	0.001	<0.001	0.224	0.041	0.536
IgA, mg/L	<0.001	<0.001	<0.001	0.029	0.196	0.640	0.423
IgM, mg/L	<0.001	0.190	<0.001	0.223	0.221	0.220	0.453
IgG, g/L	0.264	<0.001	0.008	0.705	0.722	0.573	0.503

^1^ Flock: Dorper, Katahdin-A, Katahdin-B, and St. Croix; Year: 1, 2, and 3; RG (Resistance group): High, Moderate, and Low; ^2^ ADG = average daily gain; lnFEC = logarithmic transformed fecal egg count [log(meanFEC + 100)]; PCV = packed cell volume.

**Table 8:** Effects of breed, year, and resistance group on growth and response to artificial infection with *Haemonchus contortus* by growing male sheep.

		Flock					Year				Resistance Group			
Item ^1^	Year, RG ^2^	Dorper	Katahdin-A	Katahdin-B	St. Croix	SEM	1	2	3	SEM	High	Moderate	Low	SEM
ADG, g		321 ^a^	310 ^a^	288 ^b^	269 ^b^	7.2	318 ^a^	292 ^b^	281 ^b^	6.2	303	299	290	6.2
	1	321 ^a^	317 ^a^	326 ^a^	307 ^ab^	12.4								
	2	318 ^a^	327 ^a^	263 ^bc^	259 ^c^									
	3	323 ^a^	287 ^b^	274 ^bc^	241 ^c^									
Mean FEC, eggs/g		2186 ^c^	1843 ^b^	2694 ^c^	1235 ^a^	106.1	2150 ^b^	2187 ^b^	1631 ^a^	91.0	712 ^a^	1946 ^b^	3311 ^c^	89.8
	1	3288 ^c^	1528 ^ab^	2414 ^b^	1371 ^a^	181.3								
	2	1956 ^b^	1759 ^a^	3874 ^c^	1160 ^a^									
	3	1315 ^ab^	2241 ^b^	1793 ^b^	1175 ^a^									
	High	1170 ^bc^	382 ^a^	935 ^b^	362 ^a^	178.0								
	Moderate	2172 ^d^	1437 ^c^	3045 ^def^	1128 ^bc^									
	Low	3217 ^ef^	3709 ^f^	4102 ^f^	2216 ^de^									
Mean PCV, %		29.3 ^b^	27.7 ^c^	27.9 ^c^	30.2 ^a^	0.22	29.8 ^a^	28.2 ^b^	28.4 ^b^	0.18	29.9 ^a^	28.5 ^b^	27.9 ^c^	0.18
	1	30.1 ^b^	29.3 ^bc^	28.5 ^c^	31.3 ^a^	0.37								
	2	28.4 ^cd^	27.6 ^d^	27.2 ^d^	29.6 ^b^									
	3	29.3 ^bc^	26.1 ^d^	28.2 ^cd^	29.8 ^b^									
	High						30.6 ^a^	30.0 ^ab^	29.2 ^c^	0.31				
	Moderate						29.6 ^b^	27.8 ^de^	28.2 ^d^					
	Low						29.2 ^bc^	26.8 ^e^	27.7 ^de^					
IgA, µg/mL		28.3 ^c^	50.5 ^b^	21.4 ^c^	83.0 ^a^	7.78	68.2 ^a^	36.1 ^b^	33.1 ^b^	6.74	55.3 ^a^	33.0 ^b^	49.1 ^ab^	6.68
	1	59.6 ^bc^	56.6 ^bcd^	20.3 ^d^	136.4 ^a^	13.28								
	2	11.9 ^d^	33.2 ^cd^	17.5 ^d^	81.7 ^b^									
	3	13.4 ^d^	61.7 ^bc^	26.4 ^d^	30.8 ^c^									
IgM, µg/mL		981 ^b^	950 ^b^	1223 ^a^	1276 ^a^	62.9	1174	1103	1045	54.4	1162	1136	1024	54.0
	1	846 ^c^	1208 ^b^	1473 ^ab^	1171 ^b^	107.4								
	2	1225 ^b^	660 ^c^	978 ^bc^	1550 ^a^									
	3	872 ^c^	981 ^bc^	1219 ^b^	1108 ^b^									
IgG, mg/mL		8.5	8.6	9.6	9.8	0.58	5.7 ^b^	10.4 ^a^	11.3 ^a^	0.50	9.4	9.0	9.0	0.50
	1	4.5 ^e^	7.0 ^cd^	5.4 ^de^	6.0 ^cde^	0.99								
	2	8.9 ^bc^	9.6 ^ab^	10.6 ^ab^	12.6 ^a^									
	3	12.2 ^a^	9.3 ^b^	12.9 ^a^	10.7 ^ab^									

^1^ ADG = average daily gain, FEC = logarithmic transformed fecal egg count [log(meanFEC + 100)] was used for analysis and results were presented in the original scale, PCV = packed cell volume. ^2^ RG = resistance group; IgA, IgM, and IgG = immunoglobulin A, M, and G, respectively. ^a–f^ Means within a grouping (i.e., flock, year, flock × year, resistance group, flock × resistance group, year × resistance group) without a common superscript letters differ (*p* < 0.05).

**Table 9:** Descriptive statistics of growth (average daily gain) and response to artificial infection with *Haemonchus contortus* of growing male goats and sheep from the southcentral USA in a central performance test.

Item ^1^	n	Mean	SD	Minimum	Maximum	CV
Goats						
Average daily gain, g	147	159.0	85.04	−1.0	358.8	0.53
Mean FEC, eggs/g	147	1615	1430.7	0	6788	0.89
lnFEC	147	7.10	0.909	4.61	8.84	0.13
Mean PCV, %	147	26.1	3.43	18.4	37.8	0.13
IgA, µg/mL	147	37.8	16.98	4.9	95.2	0.45
IgM, µg/mL	147	1046	445.6	251	2034	0.43
IgG, mg/mL	147	9.10	3.431	1.32	15.81	0.38
Sheep						
Average daily gain, g	200	299.6	57.21	10.2	431.9	0.19
Mean FEC, eggs/g	200	1623	1374.2	0	8250	0.85
lnFEC	200	7.07	0.989	4.61	9.03	0.14
Mean PCV, %	200	29.0	2.81	21.9	36.7	0.10
IgA, µg/mL	200	46.5	56.37	2.1	375.8	1.21
IgM, µg/mL	200	1117	422.9	126	2290	0.38
IgG, mg/mL	200	8.90	4.194	1.36	23.28	0.47

^1^ lnFEC = logarithmic transformed fecal egg count [log(meanFEC + 100)]; PCV = packed cell volume; IgA, IgM, and IgG = immunoglobulin A, M, and G, respectively.

**Table 10:** Genetic ($\sigma_{a}^{2}$), residual ($\sigma_{e}^{2}$), and phenotypic ($\sigma_{p}^{2}$) variance and heritability ($h^{2}$) estimates with standard error (SE) for average daily gain and response to artificial infection with *Haemonchus contortus* of growing male goats and sheep from the southcentral USA in a central performance test.

	Genetic Parameter							
Trait ^1^	$\sigma_{a}^{2}$	SE	$\sigma_{e}^{2}$	SE	$\sigma_{p}^{2}$	SE	$h^{2}$	SE
Goats								
ADG	1777	902.8	1894	723.5	3670	470.7	0.484	0.214
Mean lnFEC	0.256	0.2041	0.559	0.1861	0.815	0.1022	0.314	0.237
Mean PCV	4.86	2.029	3.31	1.561	8.17	1.067	0.595	0.206
IgA	59.8	42.57	173.9	40.47	233.7	28.52	0.256	0.172
IgM	33,161		65,842		99,004		0.335	
IgG	0.865	1.2110	5.34	1.252	6.20	0.737	0.139	0.192
Sheep								
ADG	2295	595.6	414	405.9	2709	309.4	0.847	0.157
Mean lnFEC	0.184	0.1606	0.722	0.1615	0.906	0.0937	0.203	0.172
Mean PCV	1.08	0.887	3.51	0.828	4.59	0.485	0.235	0.185
IgA	1191	447.0	1142	368.7	2334	249.6	0.511	0.167
IgM	87,241		73,529		160,770		0.543	
IgG	3.72	2.415	8.20	2.179	11.9	1.26	0.312	0.190

^1^ ADG = average daily gain; lnFEC = logarithmic transformed fecal egg count [log(meanFEC + 100)]; PCV = packed cell volume; IgA, IgM, and IgG = immunoglobulin A, M, and G, respectively.

**Table 11:** Estimates of genetic (below diagonal) and phenotypic (above diagonal) parameter correlations with *p* values for average daily gain and response to artificial infection with *Haemonchus contortus* of growing male goats and sheep from the southcentral USA in a central performance test.

Trait ^1^	ADG	*p* Value	lnFEC ^1^	*p* Value	PCV	*p* Value	IgA	*p* Value	IgM	*p* Value	IgG	*p* Value
Goats												
ADG			−0.049	0.556	−0.082	0.323	−0.025	0.764	−0.247	0.003	−0.317	<0.001
lnFEC	0.088	0.289			−0.155	0.061	−0.014	0.866	−0.115	0.165	−0.207	0.012
PCV	0.304	<0.001	−0.407	<0.001			0.113	0.173	−0.062	0.456	0.053	0.524
IgA	0.656	<0.001	0.388	<0.001	0.579	<0.001			0.193	0.019	0.251	0.002
IgM	−0.905	<0.001	−0.369	<0.001	−0.102	0.219	−0.573	<0.001			0.427	<0.001
IgG	−0.943	<0.001	−0.284	<0.001	−0.184	0.026	−0.603	<0.001	0.992	<0.001		
Sheep												
ADG			0.015	0.833	0.117	0.099	−0.099	0.163	−0.108	0.128	−0.227	0.001
lnFEC	0.090	0.205			−0.252	<0.001	−0.056	0.431	−0.124	0.080	−0.136	0.055
PCV	0.643	<0.001	0.205	0.004			0.138	0.051	0.155	0.028	0.019	0.789
IgA	−0.317	<0.001	0.055	0.439	0.501	<0.001			0.024	0.736	0.129	0.069
IgM	−0.178	0.012	−0.732	<0.001	0.114	0.108	0.551	<0.001			0.269	<0.001
IgG	−0.599	<0.001	−0.702	<0.001	−0.251	<0.001	0.490	<0.001	0.888	<0.001		

^1^ ADG = average daily gain; lnFEC = mean logarithmic transformed fecal egg count [log(meanFEC + 100)]; PCV = mean packed cell volume; IgA, IgM, and IgG = immunoglobulin A, M, and G, respectively.

## Data Availability

The data presented in this study are available on request from the corresponding author.
